# Matrix metalloproteinase 9 (MMP9) limits reactive oxygen species (ROS) accumulation and DNA damage in colitis-associated cancer

**DOI:** 10.1038/s41419-020-02959-z

**Published:** 2020-09-17

**Authors:** Lewins Walter, Brandon Canup, Adani Pujada, Tien Anh Bui, Behafarin Arbasi, Hamed Laroui, Didier Merlin, Pallavi Garg

**Affiliations:** 1 Institute for Biomedical Sciences, Georgia State University, Atlanta, GA United States; 2 Department of Chemistry, Georgia State University, Atlanta, GA United States; 3 Department of Biology, Georgia State University, Atlanta, GA United States

**Keywords:** Rectal cancer, Mechanisms of disease

## Abstract

Colitis-associated cancer (CAC) is a subtype of colon cancer that is driven by chronic inflammation and is prevalent in chronic ulcerative colitis patients. The development of CAC is associated with the inflammation-dysplasia-carcinoma pathway which is significantly different than adenoma-carcinoma pathway of sporadic colon cancer (CRC). Matrix Metalloproteinase 9 (MMP9) is a zinc-dependent endopeptidase against extracellular matrix (ECM) proteins expressed in the gastrointestinal tract during inflammation. We have previously shown that MMP9 plays a tumor suppressor role in CAC via “MMP9-Notch1-ARF-p53 axis” pathway. The aim of this study is to determine the role of MMP9 in maintaining genomic stability in CAC. Homozygous transgenic mice with constitutive-expression of MMP9 in the colonic epithelium (TgM9) with their wild-type littermates (WT) and stably transfected HCT116 cells with/without MMP9 were used for in vivo and in vitro experiments, respectively. As ‘proof of concept’ model, nanoparticles (NPs) loaded with MMP9 siRNA were used to examine the effect of MMP9 silencing in the colonic epithelium. In CAC, colonic epithelium of TgM9 mice exhibited lower amounts of reactive oxygen species (ROS), less DNA damage, and increased expression of mismatch repair genes compared to WTs. Our study showed that MMP9 expression correlates with the reduced ROS levels, decreased DNA damage, and upregulated mismatch repair pathway. This suggests that MMP9 expression is a natural biological way to suppress CAC by limiting ROS accumulation and DNA damage in the colon. Therefore, MMP9 inhibition could be deleterious for CAC patient.

## Introduction

Inflammatory Bowel Disease (IBD), which includes both Crohn’s Disease (CD) and ulcerative colitis (UC), is chronic inflammatory condition affecting the gastrointestinal (GI) tract^1^. IBD causes epithelial barrier disruption of the GI tract and dysregulates the major components of the GI tract such as epithelial cells, goblet cells, immune cells, and intestinal microbial populations^2^. CD affects any site in the GI tract from mouth to anus, mostly the terminal ileum, while UC occurs predominately at the distal end of the colon^3^. IBD affects approximately 1 to 4 million people in the United States^1^. Individuals with chronically active UC have up to a 50% (depending on population cohort) risk of developing colitis-associated cancer (CAC), which is driven primarily by distal colon and rectal tumors^4^. Inflammation is a beneficial response to tissue damage and pathogenic challenges. However, unregulated inflammation can be chronic resulting in malignant cell transformation and cancer^5^.

Sporadic colon cancer (CRC) and CAC are both colon cancers. However, several features make CAC distinct from CRC^6,7^. CAC and CRC have different molecular events including initiation, promotion, and progression of tumorigenesis. The development of CAC is associated with the progressive stages of dysplasia which is significantly different compared to the adenoma-carcinoma pathway of CRC progression. Unlike CRC that arise from adenomatous polyps, CAC develops commonly in flat dysplastic tissues among the individuals pre-exposed to UC and polyps are mainly localized in the distal colon region^4^.

In CAC, pro-inflammatory cytokines promote the accumulation of reactive oxygen species (ROS)^5^. Accumulation of ROS cause DNA double strand breaks (DSBs) resulting in DNA damage followed by dysregulated DNA damage response (DDR), and thereby impair the DNA repair pathways^8^. Dysregulated DDR affects the cell cycle homeostasis resulting in the proliferation of the cells with damaged DNA^9^. This results in the genomic instability due to ROS accumulation triggering the tumorigenesis^5^.

Matrix Metalloproteinases (MMPs) are zinc-dependent neutral endopeptidases with proteolytic activity against extracellular matrix (ECM) proteins^10^. MMPs can cleave or degrade proteins, clotting factors, chemotactic molecules, latent growth factors, cell surface receptors, and cell-cell adhesion molecules, thereby can regulate several biological processes^10^. Among 24 known MMPs, MMP9 is unique, as it is undetectable in healthy tissues and highly expressed in inflammation as well as in cancer^10,11^.

We have previously shown that constitutive expression of MMP9 in the colonic epithelium is protective and maintains epithelial integrity in CAC^11,12^. The aim of this study is to investigate the mechanism by which MMP9 regulates DNA genomic stability in CAC. We utilized MMP9 transgenic (TgM9) mice that constitutively express MMP9 in the colonic epithelium under the villin promoter to evaluate the protective role of MMP9 in regulating the genomic stability. We also evaluated the use of MMP9 neutralizing antibodies for clinical trials with UC patients, with the “proof of concept” in vivo model by silencing MMP9 expression by using MMP9 siRNA loaded nanoparticles (NPs).

## Results

### TgM9 mice exhibited reduced ROS levels and DNA damage in CAC

**Fig. 1:**
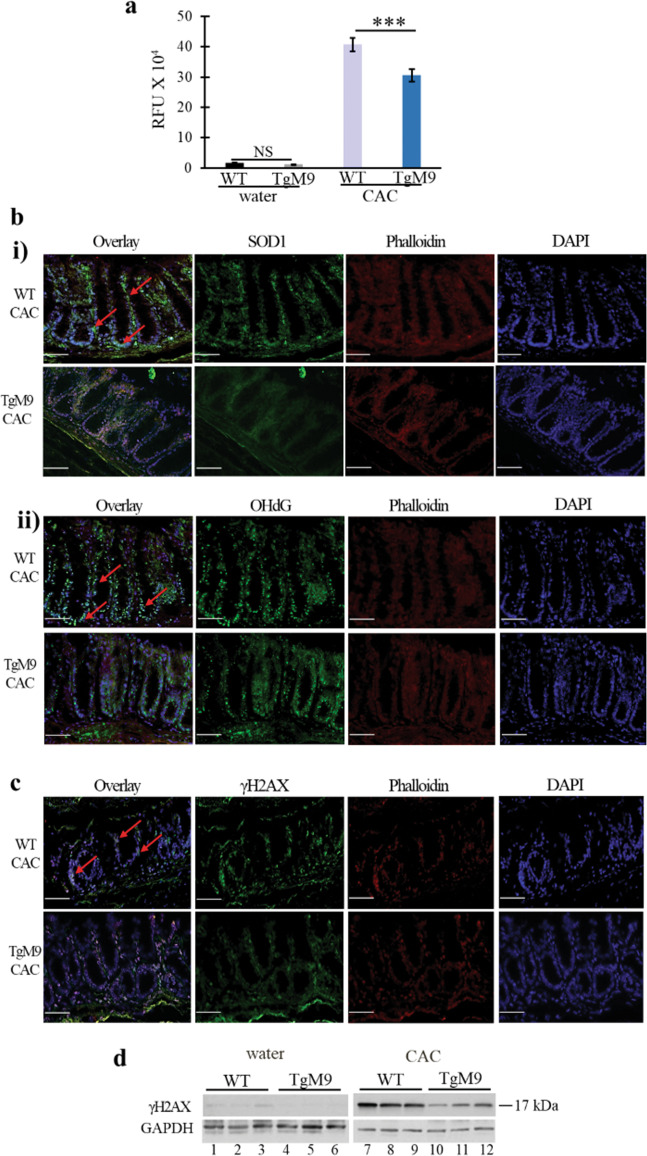
TgM9 mice exhibited reduced ROS levels and DNA damage in CAC. **a** Bar graph representation (two individual experiments) of the quantification of ROS levels in the whole colon of TgM9 and WT littermates’ mice (*n* = 10) with and without CAC. Each bar represents mean ± S.E., ***p < 0.0005 and NS is non-significant. **b** Immunofluorescence staining of the colonic Swiss rolls of TgM9 and WT littermates’ mice in CAC (*n* = 6) probed with (i) SOD1 (green fluorescence of FITC) and (ii) 8OHdG (green fluorescence of FITC). ×20 magnification was used for the images. DAPI and TRITC conjugated phalloidin were used to counterstain nuclei with blue fluorescence and actin with red fluorescence respectively. Red arrows show SOD1 and 8OHdG expressions in the colon of WT littermates’ mice. Scale bars: 50 mm. **c** Immunofluorescence staining of the colonic Swiss rolls of TgM9 and WT littermates’ mice in CAC (*n* = 6) probed with γH2AX (green fluorescence of FITC) as indicated by red arrows. ×20 magnification was used for the images. DAPI and TRITC conjugated phalloidin were used to counterstain nuclei with blue fluorescence and actin with red fluorescence respectively. Scale bars: 50 mm. **d** WB of the whole-cell lysates (25 μg/lane) extracted from mucosal stripping of TgM9 and WT littermates’ mice with and without CAC was performed and probed with anti-γH2AX. The loading control for the blot was GAPDH.

We have previously shown that MMP9 has a protective role in CAC. To understand the mechanism by which MMP9 regulates the DNA damage in CAC, we first assessed the ROS accumulation in CAC. (Fig. 1a) shows the ROS estimation of the whole colon of TgM9 mice and their wild type (WT) littermates (as control) (*n* = 10/group) in CAC, indicating the reduced ROS accumulation among the TgM9 mice compared to the control group in CAC. Interestingly, there was no significant difference in ROS levels between the TgM9 mice and their WT littermates without CAC. This quantification was also supported by the immunofluorescence staining for the two oxidative DNA damage markers. The first one is superoxide dismutase (SOD1) (as shown by red arrows in Fig. 1b-i; top panel), which utilizes free superoxide radicals and convert them to molecular oxygen and hydrogen peroxide^13^. The second one is 8-Oxo-2′-deoxyguanosine (8OHdG), which is a modified base in DNA. 8OHdG is generated by hydroxyl radicals^14^, which are produced as byproducts and intermediates during oxidative damage and aerobic metabolism (Fig. 1b-ii). Nuclear translocation of 8OHdG is an indicator (as shown by red arrows in Fig. 1b-ii, top panel) of the cellular oxidative stress. These results together suggest less oxidative stress among TgM9 mice as indicated by SOD1 (Fig. 1b-i, bottom panel) and 8OHdG (Fig. 1b-ii, bottom panel) compared to WT littermates in CAC (Fig. 1b-i and -ii, top panel respectively).

ROS accumulation causes DSBs initiating the phosphorylation of the histone variant H2AX (γH2AX) as the first major step of DNA damage^15^. The γH2AX immunofluorescence staining showed significantly lower levels of γH2AX among TgM9 mice (Fig. 1c, bottom panel) compared to WT littermates (as indicated by red arrows, Fig. 1c, top panel) in CAC. WB analysis also showed significant low protein levels of γH2AX among TgM9 mice (Fig. 1d; lanes 10–12) compared to WT mice (Fig. 1d; lanes 7–9) in CAC supporting the immunofluorescent staining results. However, there were no difference in γH2AX protein levels between the TgM9 mice and the WT littermates without CAC. These results suggest that MMP9 plays a protective role in CAC by reducing the ROS accumulation and DNA damage in the colonic epithelium.

### MMP9 expression is associated with the activation of mismatch repair (MMR) proteins in CAC

**Fig. 2:**
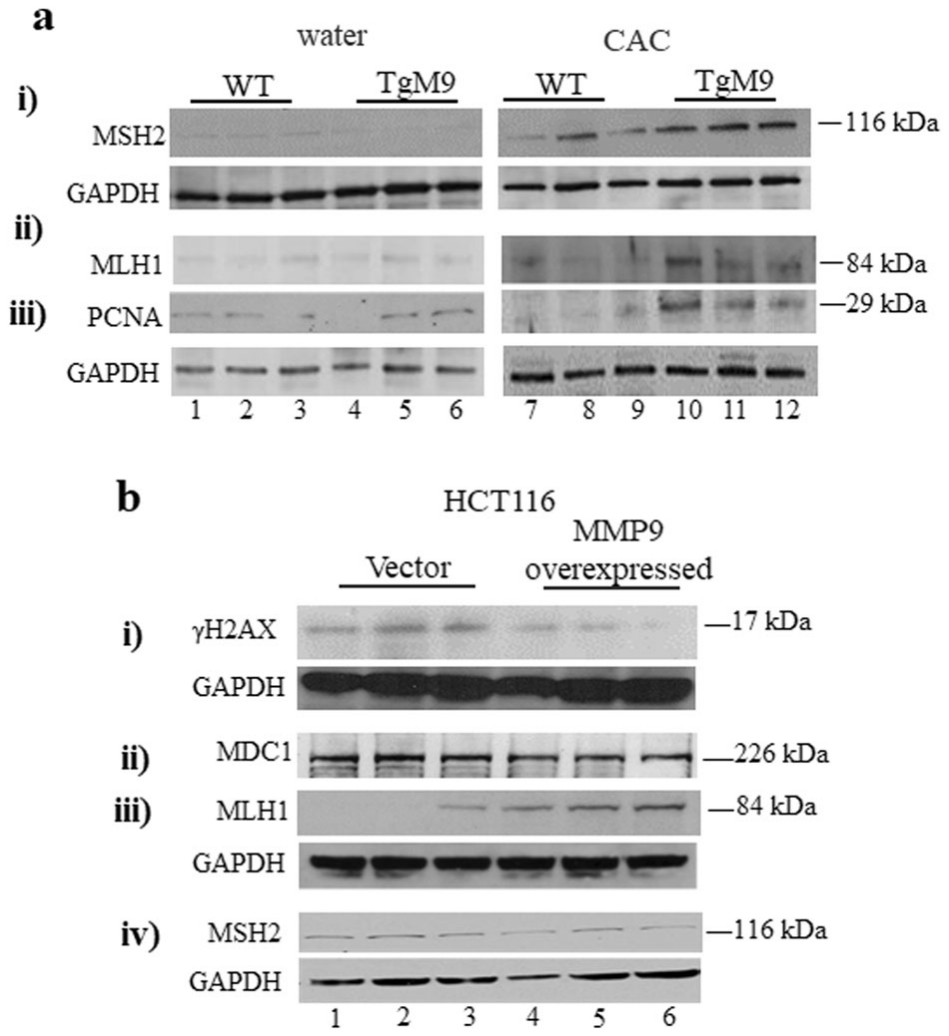
MMP9 expression is associated with the activation of mismatch repair (MMR) proteins in CAC. **a** WBs of the whole-cell lysates (25 μg/lane) extracted from mucosal stripping of TgM9 and WT littermates’ mice (*n* = 6 per group) with and without CAC were performed and probed with (i) anti-MSH2, (ii) anti-MLH1, and (iii) anti-PCNA. **b** WBs of the whole-cell lysates (25 μg/lane) of HCT116 cells with and without MMP9 expression were probed with (i) anti- γH2AX, (ii) anti-MDC1, iii) anti-MLH1, and (iv) anti-MSH2. GAPDH was used as the loading control for the blots.

Activation of MMR pathway is the defensive mechanism against DDR. ROS downregulates MMR proteins due to cytosine base methylation of MLH1 and blocks the MSH2/MSH6 dimerization^16^. To identify the role of MMP9 in MMR activation, WB analysis was performed for MMR proteins. In CAC, TgM9 mice displayed higher protein expressions of MSH2, MLH1, and pCNA (Fig. 2a; -i,-ii, -iii respectively; lanes 10–12) compared to WT littermates (Fig. 2a; -i, -ii, -iii respectively; lanes 7–9). However, there were no difference in MSH2, MLH1, and pCNA protein levels between TgM9 mice (Fig. 2a; -i, -ii, -iii respectively; lanes 4–6) and WT littermates (Fig. 2a; -i, -ii, -iii respectively; lanes 1–3) without CAC. Each blot was normalized for loading by immunoblotting with housekeeping protein GAPDH. Taken together, our results indicate that MMP9 expression promotes the activation of MMR proteins which can repair the damaged DNA in CAC.

To support the in vivo data, MMP9 expressing colon carcinoma human cell line HCT116 (stably transfected) with a vector control were used^17^. WB analysis of HCT116 cells showed that the MMP9 over expressing cells had significantly lower expressions of DNA damage marker γH2AX (Fig. 2b–i; lanes 4–6) compared to the vector control (Fig. 2a–i; lanes 1–3). A subtle decrease in MDC1, which is an adaptor protein of γH2AX^18^ in MMP9 overexpressing cells (Fig. 2b–ii; lanes 4–6) compared to vector control (Fig. 2b–ii; lanes 1–3) was also observed. Furthermore, a significant increase in mismatch repair protein MLH1 in MMP9 overexpressing cells (Fig. 2b–iii; lanes 4–6) compared to vector (Fig. 2b–iii; lanes 1–3) was observed. However, there was no significant difference for mismatch repair protein MSH2 between MMP9 overexpressing cells and the vector control (Fig. 2b–iv). Densitometry analysis of the blots has been shown in Supplementary Fig. 1. Taken together, both in vivo and in vitro data supports that MMP9 expression in colonic epithelium plays an indispensable role in limiting the DNA damage as well as promoting the activation of MMR pathway in CAC.

### Silencing of MMP9 by MMP9 siRNA loaded nanoparticles worsened CAC conditions in mice

**Fig. 3:**
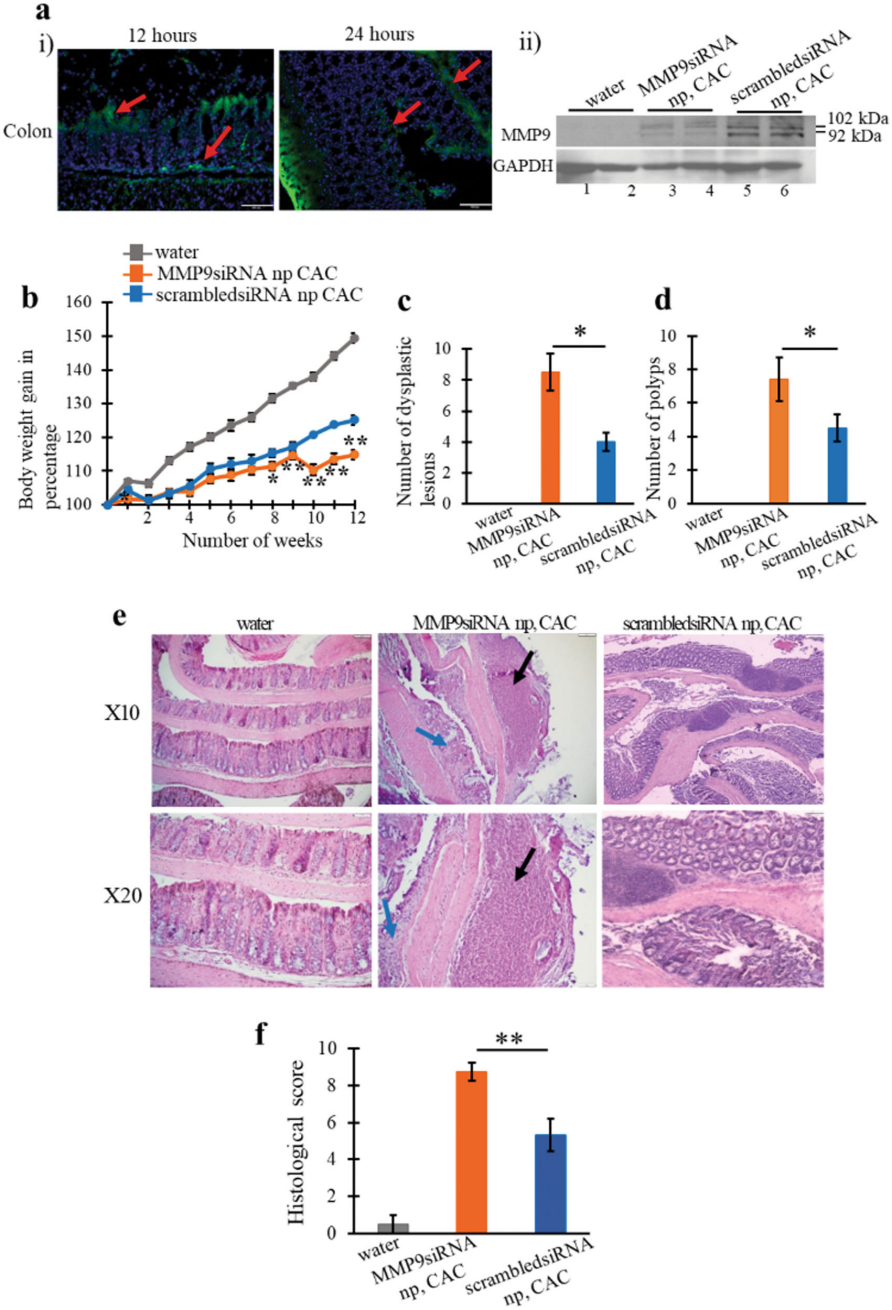
Silencing of MMP9 by MMP9 siRNA loaded nanoparticles worsened CAC conditions in mice. **a** (i) Immunofluorescence staining of nanoparticles loaded with FITC fluorescein dye as a probe after 12 and 24 h of delivery is shown by red arrows. DAPI was used to counterstain nuclei with blue fluorescence. Scale bars: 50 mm. Images were taken with ×20 magnification, and (ii) WB of the whole-cell lysates (25 μg/lane) extracted from mucosal stripping of WT mice (*n* = 5 per group, two individual experiments) with and without CAC gavaged with either MMP9 siRNA or scrambled siRNA loaded nanoparticles probed with anti-MMP9. GAPDH was used as the loading control for the blots. Bar graph presentation of the **b** body weight gain (in percent), **c** number of dysplastic lesions in the whole colon assessed under microscope, and **d** number of polyps in the whole colon assessed under microscope among WT mice (*n* = 5 per group) with and without CAC gavaged with either MMP9 siRNA or scrambled siRNA loaded nanoparticles. Each bar represents mean ± S.E., **p < 0.005 and *p < 0.05. **e** H&E staining of the colonic Swiss rolls of WT mice (*n* = 4 per group) with and without CAC gavaged with either MMP9 siRNA or scrambled siRNA loaded nanoparticles. Images were taken with ×10 and ×20 magnifications. Black arrows indicate flat polyp and blue arrows indicate dysplasia in epithelium respectively. Scale bars: 50 mm. **f** Bar graph presentation of the histological score calculated on three parameters- infiltration of neutrophils, loss of crypt architecture, and foci of ulceration. Each bar represents mean ± S.E., *p < 0.05.

To support the hypothesis that silencing of MMP9 cannot be a therapeutic strategy for CAC patients due to its protective role in CAC. WT mice (*n* = 5) were gavaged with MMP9 siRNA loaded nanoparticles (see the supplementary section). As a ‘control group’ WT mice (*n* = 5) were gavaged with scrambled siRNA loaded nanoparticles. Mice were gavaged every alternate day starting 2nd DSS cycle (Supplementary Fig. 2). Silencing of MMP9 by nanoparticles in CAC mimics the therapeutic treatments using the strategies to inhibit MMP9 expression. The successful uptake of the nanoparticle by colonic epithelium was confirmed by the immunofluorescence staining of nanoparticles loaded with FITC fluorescein dye as a probe after 12 and 24 h of delivery, as shown (Fig. 3a-i) by red arrows. Within 12 h of treatment, there was accumulation of nanoparticle in the epithelial cells which was also observed within the 24 h treatment. This confirmed that the nanoparticles were successfully delivered and up-taken into the colon cells. Nanoparticles accumulation was first observed on the surface of the mucosal layer and absorption of the nanoparticle can be observed in the 24 h treatment (Fig. 3a-i). WB analysis of the colonic whole-cell lysates indicated a significant decrease in MMP9 expression (Fig. 3a-ii; lanes 3–4) confirming the efficiency of the MMP9 siRNA loaded nanoparticles compared to scrambled siRNA loaded nanoparticle (Fig. 3a-ii, lanes 5–6) in CAC as well as to WT mice with water and no nanoparticle gavages (Fig. 3a-ii, lanes 1–2). The efficiency of the targeted delivery of nanoparticles were also examined in other organs such as small intestine (Supplementary Fig. 3a), liver (Supplementary Fig. 3b), and spleen (Supplementary Fig. 3c) in CAC. As shown in the supplementary figure, there is accumulation of the nanoparticles in the small intestine and the liver (Supplementary Fig. 3a,b). While only trace amounts of nanoparticles were observed in the spleen (Supplementary Fig. 3c). After 24 h, higher retention of the nanoparticles is only observed in the small intestine while the liver exhibited a lower signal suggesting there is lower retention in the liver. Assessment of the body weight loss indicated that WT mice gavaged with MMP9 siRNA loaded nanoparticles suffered significant body weight loss (Fig. 3b) during the recovery period after 2nd DSS cycle, starting week 4 (Supplementary Fig. 2). Figure 3c, d show significantly higher number of dysplastic lesions (as shown by blue arrows in Fig. 3e) and polyps (as shown by black arrows in Fig. 3e) respectively, among WT mice gavaged with MMP9 siRNA (dysplastic lesions- 8.5 ± 1.2 and polyps- 7.4 ± 1.3) loaded nanoparticles compared to control group (dysplastic lesions- 4 ± 0.6 and polyps- 4.5 ± 0.8) in CAC. H&E staining in Fig. 3e indicated extensive crypt damage, increased neutrophil infiltration, more ulceration foci, and dysplastic lesions among WT mice receiving MMP9 siRNA compared to control group in CAC. Figure 3f is the bar graph presentation of the histological score (calculated on the parameters described in Methods section). The histological score (Fig. 3f) was significantly higher among WT mice gavaged with MMP9 siRNA loaded nanoparticles (8.75 ± 0.96) compared to control group (5.33 ± 1.53) in CAC. Taken together, our “proof of concept” model, shows that the suppression of MMP9 in the colon significantly worsens CAC.

### Silencing of MMP9 by nanoparticles alters the microbiota and ROS levels in CAC

**Fig. 4:**
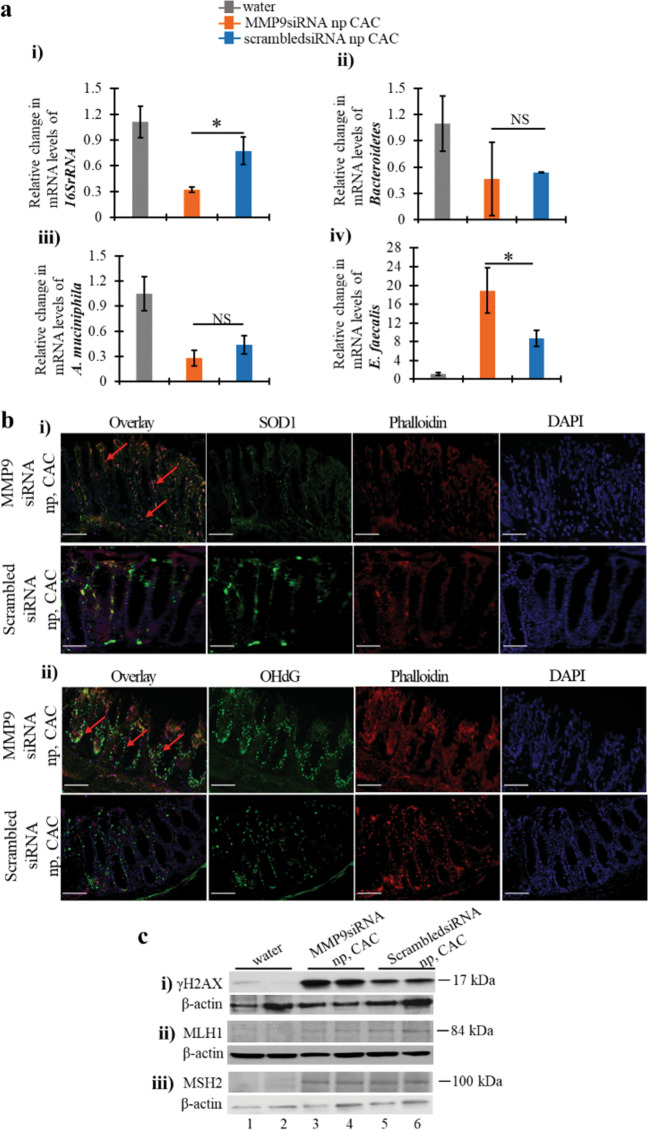
Silencing of MMP9 by nanoparticles alters the microbiota and ROS levels in CAC. **a** Bar graph presentation of the QPCR analyses of different phyla of microbiota at mRNA levels in colonic mucosal stripping from WT mice (*n* = 5 per group) with and without CAC gavaged with either MMP9 siRNA or scrambled siRNA loaded nanoparticles. Relative mRNA expression levels of (i) universal bacteria, *16SrRNA* (ii) *Bacteroidetes* (iii) *A. muciniphila* (iv) *E. faecalis*. NS means non-significant. Each bar represents mean ± S.E., *p < 0.05. **b** Immunofluorescence staining of the colonic Swiss rolls of WT mice (*n* = 5 per group) gavaged with either MMP9 siRNA or scrambled siRNA loaded nanoparticles in CAC probed with (i) SOD1 (green fluorescence of FITC) and (ii) 8OHdG (green fluorescence of FITC). ×20 magnification was used for the images. DAPI and TRITC conjugated phalloidin were used to counterstain nuclei with blue fluorescence and actin with red fluorescence respectively. Red arrows show the increased staining of SOD1 and 8OHdG in the colon of WT mice gavaged with MMP9 siRNA loaded nanoparticles in CAC. Scale bars: 50 mm. **c** WBs of the whole-cell lysates (25 μg/lane) extracted from mucosal stripping of WT mice (*n* = 5 per group) with and without CAC gavaged with either MMP9 siRNA or scrambled siRNA loaded nanoparticles were performed and probed with (i) anti- γH2AX, (ii) anti-MLH1, and (iii) anti-MSH2. β-actin was used as the loading control for the blots.

Colonic inflammation causes alterations in the microbiota homeostasis triggering cancer progression^12,19^. The changes in microbial population due to MMP9 silencing were next investigated by qPCR (Fig. 4a). There was a significant decrease in mRNA levels for *16SrRNA* (universal bacteria) among WT mice gavaged with MMP9 siRNA loaded nanoparticles (0.32 ± 0.03 fold) versus control group (0.77 ± 0.15 fold) in CAC (Fig. 4a-i). However, there were no significant difference in the mRNA levels for *Bacteroidete*s (Fig. 4a-ii) and *Akkermansia muciniphila* (Fig. 4a-iii) between the WT mice gavaged with either MMP9 siRNA or scrambled siRNA loaded nanoparticles in CAC. Interestingly, WT mice gavaged with MMP9 siRNA loaded nanoparticles (18.89 ± 4.81 fold) showed significant increase in mRNA levels for *Enterococcus fecalis* compared to control group (8.72 ± 1.74 fold) in CAC.

In chronic inflammation disruption of microbiota homeostasis and ROS generation form an auto feedback loop^8,20,21^ and thereby propagates the progression of inflammation. We assessed the effect of MMP9 silencing on ROS production by immunofluorescence staining of the oxidative DNA damage markers 8OHdG (Fig. 4b-i) and SOD1 (Fig. 4b-ii). There were higher ROS levels among WT mice gavaged with MMP9 siRNA compared to the WT mice gavaged with scrambled siRNA/ control group (as shown by red arrows in Fig. 4b) in CAC. WB analysis was performed to analyze the DNA damage and activation of MMR proteins. Figure 4c-i shows increased protein levels of DNA damage marker γH2AX among WT mice gavaged with MMP9 siRNA (lanes 3–4) compared to the control group (lanes 5–6). It was also observed that silencing of MMP9 by siRNA in WT mice resulted in the decreased protein levels of MLH1 (Fig. 4c-ii, lanes 3–4) compared to the control group (Fig. 4c-ii, lanes 5–6) in CAC. However, there was no difference in MSH2 protein levels between the WT mice gavaged with either MMP9 siRNA (Fig. 4c-iii, lanes 3–4) or scrambled siRNA (Fig. 4c-iii, lanes 5–6). Densitometry analysis of the blots has been shown in Supplementary Fig. 4. These results together imply that MMP9 expression is necessary to prevent DNA damage as well as for the activation MMR proteins in CAC.

### MMP9 expression promotes epithelial cells differentiation and proliferation

**Fig. 5:**
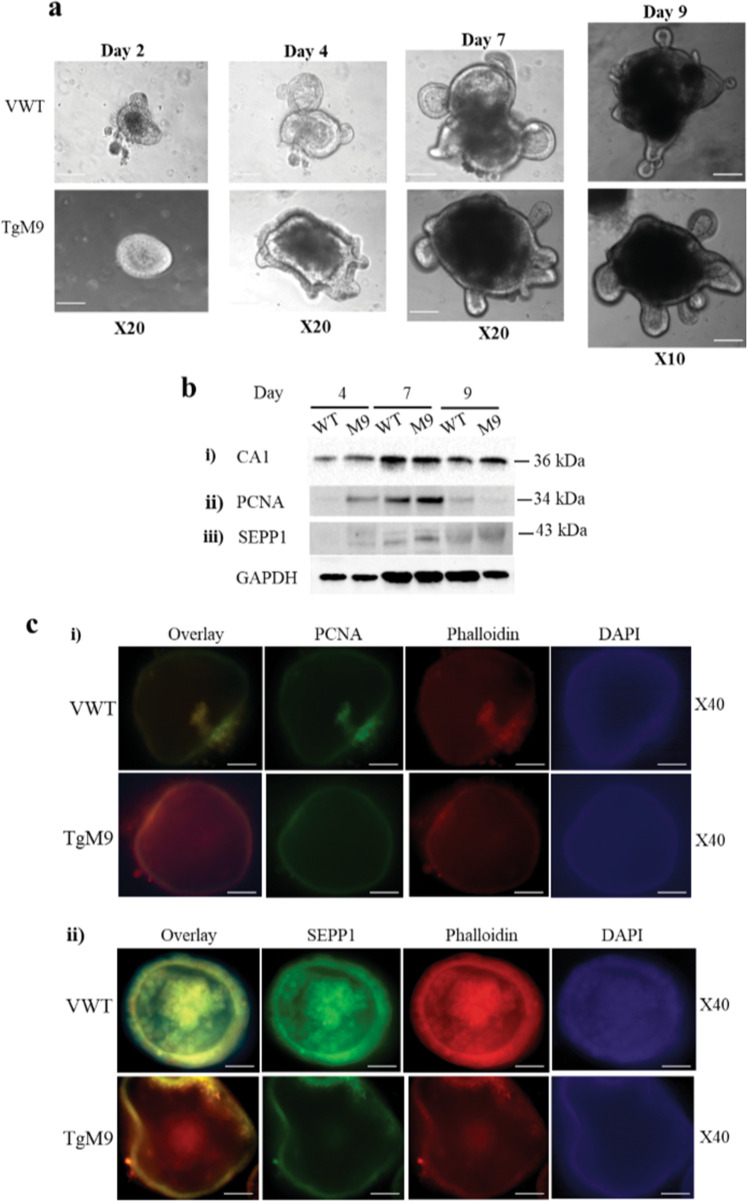
MMP9 expression promotes epithelial cells differentiation and proliferation. **a** Crypts isolated from the colons of TgM9 and their WT littermates’ mice were grown to form colonoids on Matrigel in 24 well plates. Colonoid formation was observed and images were captured on (i) day 2, (ii) day 4, (iii) day 7 at ×20 magnification, and (iv) day 9 at ×10 magnification. Scale bars: 50 mm. **b** WBs of the whole-cell lysates of the colonoids from TgM9 and their WT littermates’ mice (10 μg/lane for day 4 and 20 μg/lane for days 7 & 9) were probed with (i) anti- CA1, (ii) anti-SEPP1, and iii) anti-PCNA. GAPDH was used as the loading control for the blots. Each blot is a representation of three individual experiments. **c** Immunofluorescence staining of four days old colonoids of TgM9 and their WT liitermates’ mice, grown on Matrigel in chambered slides (*n* = 4 wells per group) probed with (i) PCNA (green fluorescence of FITC) and (ii) SEPP1 (green fluorescence of FITC). ×40 magnification was used for the images. DAPI and TRITC conjugated phalloidin were used to counterstain nuclei with blue fluorescence and actin with red fluorescence respectively. Images are the Z-sections with ×40 magnification. DAPI was used to counterstain nuclei with blue fluorescence and red fluorescence is for actin-phalloidin. Scale bars: 50 mm.

Loss of normal and healthy epithelium escalates CAC progression^22,23^. To investigate the role of MMP9 in preventing the loss of healthy epithelium, crypts were extracted from TgM9 and WT littermates’ mice on Matrigel (as described in the Method section) and were used as an ex vivo model. The crypts growing into the colonoids were followed for days 2, 4, 7, and 9 (Fig. 5a, i–iv respectively). Crypts isolated from TgM9 mice differentiated faster (Fig. 5a-ii) and proliferated earlier (Fig. 5a-iii and Fig. 5a-iv) to form the colonoids compared to the crypts isolated from the WT littermates. WB was also performed to assess the differentiation and proliferation. Figure 5b-i showed higher protein levels of carbonic anhydrase-1 (CA1)^24^, which is an enterocyte marker among TgM9 mice derived colonoids on day 4 and day 9 compared to the colonoids derived from WT littermates’ mice. Figure 5b-ii indicated increased protein levels of the proliferation marker PCNA^25^ on days 4 and 7 among TgM9 mice derived colonoids. Interestingly, WB data showed that once colonoids were completely differentiated and proliferated, PCNA expression went down among TgM9 mice derived colonoids compared to WT littermates’ mice (Fig. 5b-ii). Figure 5b-iii showed increased expression of selenoprotein P (SEPP1) an antioxidant marker^26^ among the TgM9 mice derived colonoids compared to WT littermates’ mice for days 4, 7, and 9 respectively. Densitometry analysis has been shown in Supplementary Fig. 5. Immunofluorescence staining for PCNA (Fig. 5c-i, top panel) indicated more PCNA positive cells among four days old colonoids derived from TgM9 mice compared to WT littermates’ mice, supporting the WB data. Similarly, immunofluorescence staining for SEPP1 (Fig. 5c-ii, bottom panel) indicated higher SEPP1 expression among four days old colonoids derived from TgM9 mice compared to WT littermates’ mice. The ex vivo data with colonoids imply that MMP9 plays a role in maintaining the differentiation and proliferation of crypts in colonic epithelium.

## Discussion

The risk of developing CAC among the IBD affected individuals is mainly confined to UC patients^27,28^. In CAC, inflamed epithelial cells become dysplastic and gradually advance through the stages of low to high-grade dysplasia and is therefore also referred as dysplasia driven colorectal cancer^6,29^. Chronic inflammation in response to an infection or tissue damage causes oxidative stress due to the recruitment of immune cells and inflammatory mediators^5^. Oxidative stress is a key component of chronic inflammation due to the release of ROS into the tissue environment^30^. Accumulation of ROS causes initiation and progression of tumorigenesis due to the oxidative damage of DNA followed by the activation of the oncogenes^31^. The overwhelming DNA damage are beyond the restoration capacity of the DNA repair pathways^32,33^. This results in genomic instability, which fosters the dysplasia and cancer progression. Genomic instability is marked by abnormal cell cycle, deactivation of tumor suppressors, or failure of MMR pathways^5,34^.

**Fig. 6:**
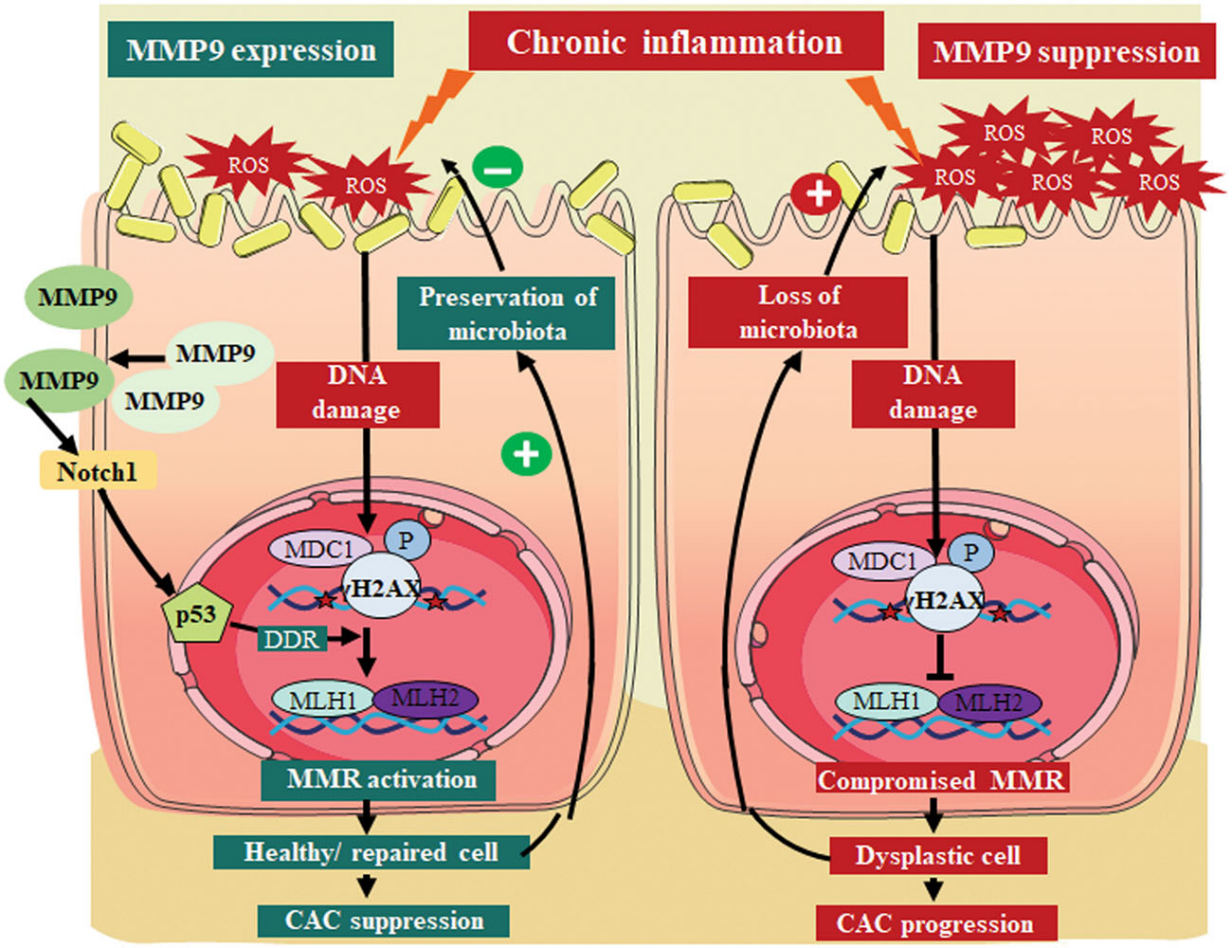
Schematic presentation of MMP9 mediated inhibition of reactive oxygen species (ROS) accumulation. In CAC, chronic inflammation generates ROS as well as activates MMP9. Active MMP9 is secreted out in extracellular space. MMP9 upregulates p53 expression via activation of transmembrane protein Notch1^11^. Activation of p53 stimulates DNA damage response (DDR) to prevent ROS mediated DNA damage. DDR causes activation of mismatch repair (MMR) proteins to repair damaged DNA. This prevents the dysplasia progression and allows the proliferation of healthy epithelium. Preservation of healthy epithelium ensures the microbiota homeostasis and regulates ROS production. These events act as a positive feedback loop triggered by MMP9 causing the CAC suppression. Silencing of MMP9 accelerates CAC progression due to an auto feedback loop set up by the loss of microbiota and increase in ROS accumulation. This results in epithelial cell dysplasia in CAC.

Being a secretory proteinase, MMP9 is one of the essential regulators of ECM and basement membrane (BM)^10,35,36^. We have previously reported the tumor suppressor role of MMP9 in CAC^17,37^ showing that MMP9 activates Notch1-p53-ARF pathway^11^ and promotes epithelial integrity^12^ in CAC. In this study, we investigated the role of MMP9 in maintaining genomic stability in CAC. Our in vivo data with transgenic mice that constitutively express MMP9 in the colonic epithelium suggest that MMP9 expression is associated with lower levels of ROS in CAC as indicated by the ROS markers, 8OHdG and SOD1. Lower ROS accumulation prevents DNA damage in CAC as evident by the DNA damage marker, γH2AX. We also observed MMP9 mediated activation of MMR proteins (MLH1 and PCNA) in CAC. Our in vitro data with human colonic cells HCT116 corroborated the in vivo findings. MMP9 silencing in colonic epithelium by MMP9 siRNA loaded nanoparticles, as the ‘proof of concept’ model indicate that silencing of MMP9 expression exacerbate CAC conditions in mice causing weight loss, more polyps, dysplastic lesions, higher ROS, and excessive DNA damage. Higher ROS levels and dysplastic epithelium resulted in dysregulated microbiota among mice in CAC. Using colonoids as ex vivo model, we observed that MMP9 regulates differentiation and proliferation to maintain epithelial homeostasis. Taken together, these results mechanistically elucidate the tumor-suppressive role of MMP9 in CAC by promoting genomic stability (Fig. 6). Our study implies that MMP9 expression by colonic epithelial cells is necessary for CAC suppression. Our study also emphasizes that silencing of MMP9 will result in the CAC aggravation due to the loss of microbiota homeostasis and ROS accumulation, which together results in dysplasia progression (Fig. 6).

Chronic inflammation induced ROS accumulation acts as a double edge sword in CAC. On one hand, it causes DNA damage and triggers the oncogene mutations resulting in dysplasia initiation. On the other hand, ROS accumulation depletes the microbiota and exposes the inflamed/dysplastic tissue to inflammatory environment and thereby promoting the dysplasia progression.

MMP9 associated downregulation of SOD1, 8OHdG, and γH2AX signifies the lower ROS in CAC. Due to oxidative stress, guanosine is oxidized to 8OHdG which induces gene transversions causing p53 mutations^14^, which are the first molecular events in CAC tumorigenesis^6^. MMP9 mediated lower expression of γH2AX suggests decrease oxidative DNA damage. H2AX a variant of histone 2A of the DNA is phosphorylated at the DNA breaks during DNA damage, and serves as a docking site for DNA repair proteins^15^. Therefore, γH2AX is a direct measure of DSBs. MMP9 associated activation of MMR proteins is the DNA damage response to maintain the genomic stability in CAC. Activation of MMR proteins are very critical to correct the replication of damaged DNA^16^. These together elucidate the underlying mechanism by which MMP9 maintains the genomic instability in CAC. Prevalence of *E. faecalis* is an index of chronic inflammatory response, promoting the ROS production^38^. It is also known to impair DNA repair pathways^38^. Therefore, MMP9 expression correlating with the lower abundance of *E. faecalis* is a significant evidence of MMP9’s role in maintaining the genomic stability in CAC.

Loss of healthy epithelium favors tumorigenesis. MMP9 expressing crypts displayed higher expression of CA1 and PCNA. CA1 is the marker of epithelial differentiation and its higher expression signifies that MMP9 can promote the faster differentiation of epithelial cells to reinstate the healthy epithelium in CAC. Furthermore, PCNA as a proliferation marker suggests that MMP9 regulates the normal proliferation of healthy epithelial cells. MMP9 expressing crypts also displayed higher levels of SEPP1 ensuring the ROS homeostasis in epithelial cells. Regulation of differentiation as well as proliferation to maintain the homeostasis of healthy epithelium is critical in delaying or preventing the CAC progression.

There are studies showing the beneficial and protective role of MMP9 in other pathological conditions. Our study suggests that the tumor-suppressive role of MMP9 could be tissue specific and/or disease specific. MMP9 promotes liver recovery from ischemia and reperfusion injury^39^, protects in chronic kidney disease^40^, and suppresses systemic autoimmune disease^41^. The present study also clarifies the failure of the clinical trials using MMP9 antibodies or MMP9 neutralizing antibodies. These trials were based on the studies which used either sporadic colon cancer models or acute colitis models^42,43^. However, none of these models are adequately specific to study the CAC and chronic UC pathogenesis. Our ‘proof of concept’ model using MMP9 siRNA loaded nanoparticles suggests that MMP9 silencing worsens the disease conditions among CAC patients. Therefore, MMP9 inhibition should be avoided as CAC treatment. Understanding of the unique defensive role of MMP9 in CAC is important to explore the efficient therapeutics for CAC patients which will significantly decrease the healthcare costs and will improve the quality of their life.

## Materials and methods

### Animal models

All animal procedures were performed in accordance with the Guide for the Care of Use of Laboratory Animals as described previously^11^. Gender matched 10 weeks old TgM9 and their WT littermates of C57/B6 background were used in this study.

### Colitis-associated cancer induction

As described previously^17^, CAC was induced by one intraperitoneal injection (i.p.) of 7.6 mg/kg azoxymethane (AOM, Sigma, St. Louis, MO, USA). After a week, TgM9 and their WT littermates’ mice were given 3% (w/v) dextran sodium sulfate (DSS, MP Biomedicals, Solon, OH, USA), through drinking water for a week, followed by two weeks of recovery period (by switching them back to regular drinking water). On day 28, mice were given 2^nd^ DSS cycle followed by the recovery cycle and were sacrificed on day 56. Body weight, stool consistency, and stool occult blood data for all the mice were recorded during both DSS and recovery cycles^17^.

### Reactive oxygen species assay

According to the manufacturer’s instructions, freshly collected whole colon was processed using ROS assay kit (Cell Biolabs Inc., San Diego, CA, USA)^44^. Then it was analyzed with a fluorometric plate reader at 480/530 nm.

### Immunofluorescence staining of mouse tissues

As described previously^45^, immunofluorescence staining was performed with the colonic Swiss rolls of the TgM9 and their WT littermates’ mice in CAC. The primary antibodies used were anti-superoxide dismutase 1 (SOD1, Santa Cruz Biotechnology, Dallas, TX, USA), anti-8-Oxo-2′-deoxyguanosine (8OHdG, Santa Cruz Biotechnology), anti-γH2AX (Abcam, Cambridge, UK), and anti-GFP (Santa Cruz Biotechnology). The tissues were mounted with ProLong Antifade mounting medium with 4′,6-diamidino-2-phenylindole (DAPI, nuclear stain) (Thermo Fischer Scientific, Waltham, MA, USA) and were analyzed using an Olympus microscope equipped with a D-26 color camera at ×20 magnification.

### Protein extraction and western blot (WB) analysis

As described previously^11^. WB analysis was performed by using the whole-cell lysates (30 μg per well) of the colonic mucosal stripping of TgM9 and their WT littermates’ mice with and without CAC. The primary antibodies used were anti-MMP9 (Cell Signaling, Beverly, MA, USA), anti-γH2AX (Abcam), anti-MDC1 (Novus Biologicals, Littleton, CO), anti-MLH1 (Abcam), anti-MSH2 (Abcam), and anti-PCNA (Abcam). Goat anti-mouse secondary antibody (Bio-Rad, Hercules, CA) or goat anti-rabbit secondary antibody (Abcam) were used. Anti-GAPDH (Abcam) or anti-β actin (Sigma) antibodies were used as the WB loading controls. Bio-Rad Quantity One software was used for densitometry analysis.

### MMP9 siRNA/PEI-loaded NPs covered with PVA preparation

The internal phase was prepared with N/P ratio of PEI/siRNA of 30, using the number of negative charges of siRNA (MMP9 siRNA, FITC-siRNA or scrambled siRNA) (P as the phosphorous charge) and positive charges of PEI (N as the ammonium charge). A mixture of siRNA/PEI: 29 μL MMP9 or scrambled siRNA (5 μM) was combined with 18 μL PEI (5 mM) and incubated for 10 min at room temperature for complexation. After 10 min, a polyplex was formed, and 750 μL bovine serum albumin (BSA, 50 g/L) was added, generating the first emulsion with dichloromethane.

As described previously^46^, NPs were synthesized via double emulsion/solvent evaporation. The internal phase with the siRNA was mixed with 20 g/L of polylactic acid (PLA) in dichloromethane to generate a water-in-oil (W/O) emulsion after 2 min of vortexing (Maxi Mix II, Thermodyne, Dubuque, IA, USA) as well as 1 min of sonication with 50% active cycles at 70% power (Pmax = 400 W) (Digital Sonifier 450, Branson, Danbury, CT, USA). The initial emulsion was dropped in an additional water phase containing 0.3 g/L of PVA to produce a water/oil/water emulsion (W/O/W).

The W/O/W emulsion was dropped in a dispersing phase of 0.1 g/L polyvinylic alcohol (PVA) and stirred at 45 °C under a vacuum to remove dichloromethane. NPs were centrifuged at 9953 × *g* and freeze-dried overnight at −50 °C under 0.1 mbar pressure. As the second emulsion allowed PVA to be grafted on the surface by hydrophobic interaction with the PLA matrix, NPs were coated with PVA to prevent aggregations through electrostatic repulsions.

### Gavage of scrambled, MMP9 siRNA, and FITC siRNA PLA NPs

Ten weeks old both male and female WT C57BL/6 mice (12 per group) received alternate day gavages after the second DSS cycle (Supplementary Fig. 1) with either MMP9 siRNA loaded PLA NPs or scrambled siRNA loaded PLA NPs. The biodistribution was done using 1 mg/ml of total FITC NP concentration for 12 and 24-hour post 24 h fasting period. Then, mice were sacrificed, and colons were collected for biological analysis.

### Hematoxylin and Eosin staining

As described previously^11^, hematoxylin and eosin (H& E) staining of the colonic Swiss rolls of the WT mice with and without CAC gavaged with MMP9 siRNA or scrambled siRNA loaded nanoparticles were performed and analyzed using Olympus microscope (Olympus, Waltham, MA, USA)equipped with a D-26 color camera at ×20 magnification. Histological score was calculated (blindfold) on H&E stained tissues considering three parameters- infiltration of neutrophils, loss of crypt architecture and foci of ulceration.

### RNA extraction and qPCR

As described previously^12^, total RNA was extracted from colonic tissues of the WT mice with and without CAC gavaged with MMP9 siRNA or scrambled siRNA loaded nanoparticles using the RNeasy mini Kit (Qiagen, Valencia, CA, USA). mRNA expression was quantified by quantitative real-time reverse-transcription polymerase chain reaction using Maxima SYBR green quantitative polymerase chain reaction Master Mix (Thermo Scientific). Forward and reverse primer sequences for *16SrRNA*, *Bacteroidete*s, *A. muciniphila*, and *E. faecalis* are listed in the Supplementary Table 1.

### Cell culture and transfection

As described previously^11^, stably transfected human colonic HCT116 cells were used. Briefly, they were transfected with a pEGFP plasmid with and without the MMP9 gene and were selected under the antibiotic Geneticin (GIBCO, Grand Island, NY, USA). These transfected clones were screened for MMP9 expression by WB and the three highest MMP9 expressing clones were selected for HCT116 cell line and were sorted via flow cytometry (BD Biosciences).

### Colonoid extraction

As described by Mahe et al.^47^, crypts were isolated from six weeks old gender matched TgM9 and their WT littermates mice and were grown to colonoids on Matrigel (Corning, New York, NY, USA) in 24 well plate. Colonoids grown on 24 well plates were observed every day and pictures were taken at ×10 and ×20 magnification with ZEISS Primovert AxioCam MRm microscope for days 2, 4, 7 and, 9.

### Western blot analysis of colonoids

Colonoids of TgM9 and their WT littermates’ mice grown on Matrigel in a 24 well plate were washed with room temperature 1X PBS. Then cold 1X PBS was added to disrupt the colonoids. Disrupted colonoids were centrifuged at 5000 rpm for 5 min at 4 °C. After carefully removing and discarding the supernatant (mix of PBS and Matrigel), colonoid-pellet was lysed with cell lysis buffer RIPA^11^] and protein estimation was done. Colonoid lysates (10 µg for day 4 and 20 µg for day 7 and day 9) were used for the gel electrophoresis. The primary antibodies used were anti-CA1 (kind gift from Dr A. Waheed; Department of Medicine, St. Louis University, St. Louis, MO, USA), anti-PCNA (Abcam), anti-SEPP1 (Abcam). Goat anti-rabbit secondary antibody (Abcam) was used. Equal loading of the WBs was normalized by using anti-GAPDH as the loading control.

### Immunofluorescence staining of colonoids

TgM9 mice and their WT littermates’ colonoids were grown on Matrigel for 4 days in Tissuetek chambered slides (Thermo-Fischer) for immunofluorescence staining. As described by Wang et al.^48^, colonoids were fixed with 4% paraformaldehyde solution for 2 h and washed with 1X PBS. Colonoids were permeabilized with 2% TritonX100 solution and washed with immunofluorescence buffer (IF buffer: 0.2% TritonX100, 0.05% Tween 20 in 1X PBS). Blocking buffer (2% TritonX100, 10% goat serum, and 0.02% sodium azide in 1XPBS) was used and then, colonoids were incubated overnight with primary antibodies anti-PCNA and anti-SEPP1 (in dilution buffer (0.25% TritonX100, 1% normal serum goat, 0.02% sodium azide in 1X PBS). Colonoids were washed with IF buffer followed by incubation with secondary antibody anti-rabbit Alexa Fluor 488- green (Thermo-Fisher). After washing with IF buffer they were incubated with TRITC-phalloidin in dilution buffer for 2 h. Colonoids were washed with IF buffer and incubated with Hoechst 3342, Trihydrochloride, Trihydrate (Thermo Fischer Scientific) in dilution buffer for 1 h for nuclei staining. Z-section images (blindfold) were taken with Keyence BZ-X700 microscope at ×40 magnification.

### Statistical analysis

As described previously^11^, data are presented as means ± SE. Groups (equal or unequal variance) were compared by Student’s *t* test. *P* values < 0.05 was considered statistically significant.

## Supplementary information

Supplementary Figure Legends

Supplementary Figure 1

Supplementary Figure 2

Supplementary Figure 3

Supplementary Figure 4

Supplementary Figure 5

Supplementary Table 1
