# Supplementary material for: Matrix metalloproteinase 9 (MMP9) limits reactive oxygen species (ROS) accumulation and DNA damage in colitis-associated cancer
Source: Cell Death Dis. 2020 Sep 17;11(9):767. doi: 10.1038/s41419-020-02959-z (PMC7498454; doi:10.1038/s41419-020-02959-z)
Supplement: Supplementary file 1 — Supplementary Figure Legends [file 41419_2020_2959_MOESM1_ESM.doc]

**Supplementary Figure 1. Densitometry analysis of γH2AX, MDC1, MLH1, and MSH2 protein expressions in HCT116 cells.** Bio-Rad One software was used for the bar graph presentation of (a) γH2AX, (b) MDC1, (c) MLH1, and (d) MSH2 protein expression among HCT116 cells overexpressing MMP9 compared to vector control by normalizing the WB bands with GAPDH as loading control.

**Supplementary Figure 2. Schematic of CAC model used for the study.** CAC was induced by one intraperitoneal injection of azoxymethane (AOM) and two cycles of dextran sodium sulfate (DSS). Each DSS cycle (1 week) was followed by two weeks of recovery cycle. MMP9siRNA or scrambled siRNA loaded nanoparticles were gavaged at the beginning of 2^nd^ DSS cycle on every alternate day. End point of the experiment was day 56. Red arrows represent the start point of DSS cycle and green arrows represent the start point of recovery cycle.

**Supplementary Figure 3. Efficiency of the targeted delivery of the nanoparticles in mice.** The uptake of the nanoparticle by other organs was confirmed by the immunofluorescence staining of nanoparticles loaded with FITC fluorescein dye as a probe after 12 and 24 hours of delivery, as shown by red arrows. Within 12 hours of treatment (a) few small intestine epithelial cells exhibited nanoparticle uptake, (b) most of the liver cells exhibited nanoparticle uptake, and (c) lower uptake of nanoparticle in spleen. After 24 hours treatment most of the nanoparticles were not retained by small intestine, liver and spleen. (n=4 per experiment). Images are representative of two experiments. Scale bars: 50 mm.

**Supplementary Figure 4. Densitometry analysis of γH2AX, MLH1, and MSH2 protein expressions among WT mice gavaged with MMPsiRNA and/or scrambled siRNA.** Bio-Rad One software was used for the bar graph presentation of (a) γH2AX, (b) MLH1, and (c) MSH2 protein expressions among WT mice gavaged MMPsiRNA and/or scrambled siRNA by normalizing the WB bands with β actin as loading control.

**Supplementary Figure 5. Densitometry analysis of CA1, PCNA, and SEPP1 protein expressions among colonoids.** Bio-Rad One software was used for the bar graph presentation of (a)CA1, (b) PCNA, and (c) SEPP1 protein expressions among organoids derived from TgM9 mice and WT mice by normalizing the WB bands with GAPDH as loading control.

**Supplementary Table 1. Sequences of the QPCR primers used in the study.**
