# Supplementary figures and images for: Matrix metalloproteinase 9 (MMP9) limits reactive oxygen species (ROS) accumulation and DNA damage in colitis-associated cancer
Source: Cell Death Dis. 2020 Sep 17;11(9):767. doi: 10.1038/s41419-020-02959-z (PMC7498454; doi:10.1038/s41419-020-02959-z)

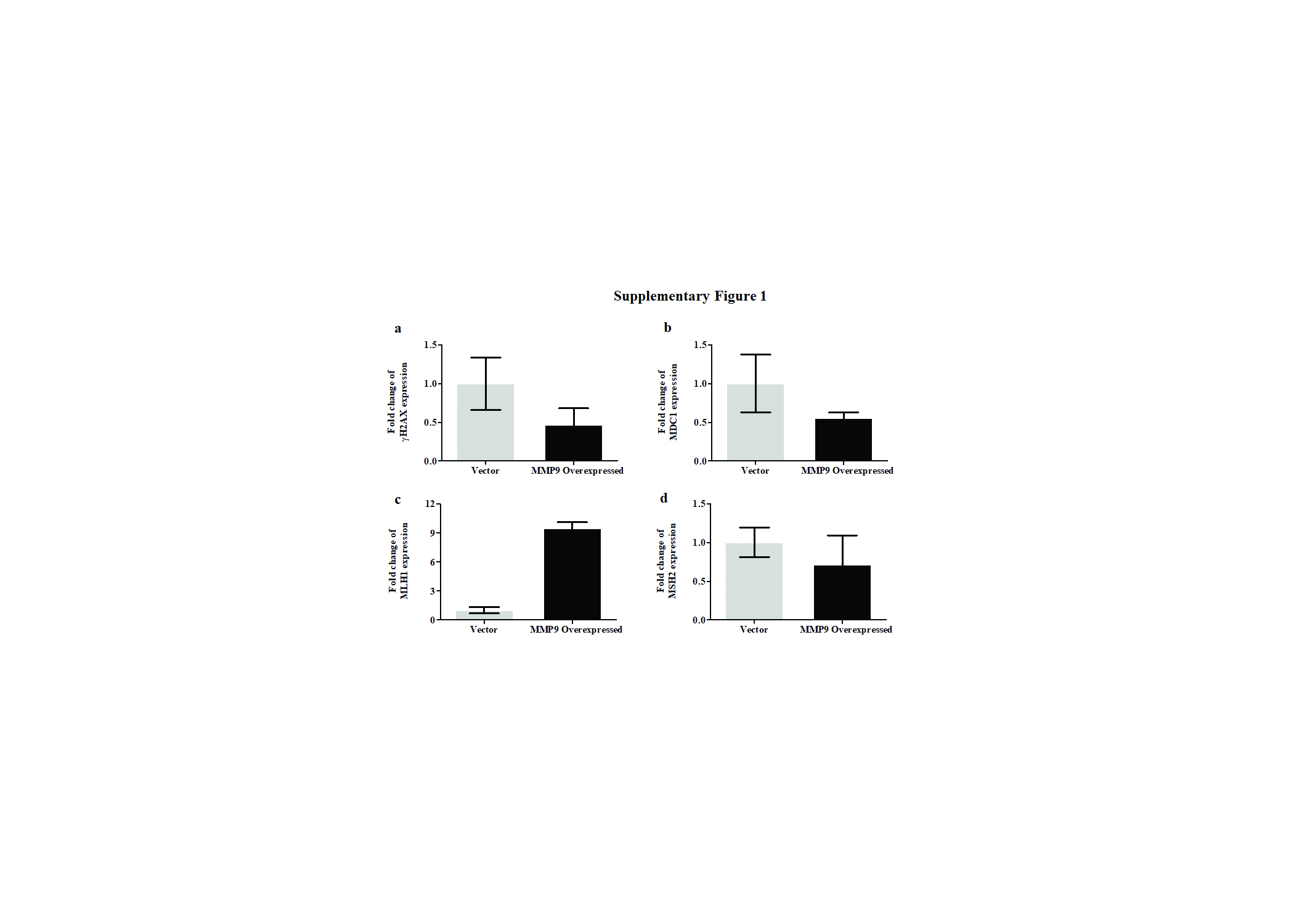

Supplement: Supplementary file 2 — Supplementary Figure 1 [file 41419_2020_2959_MOESM2_ESM.tif]

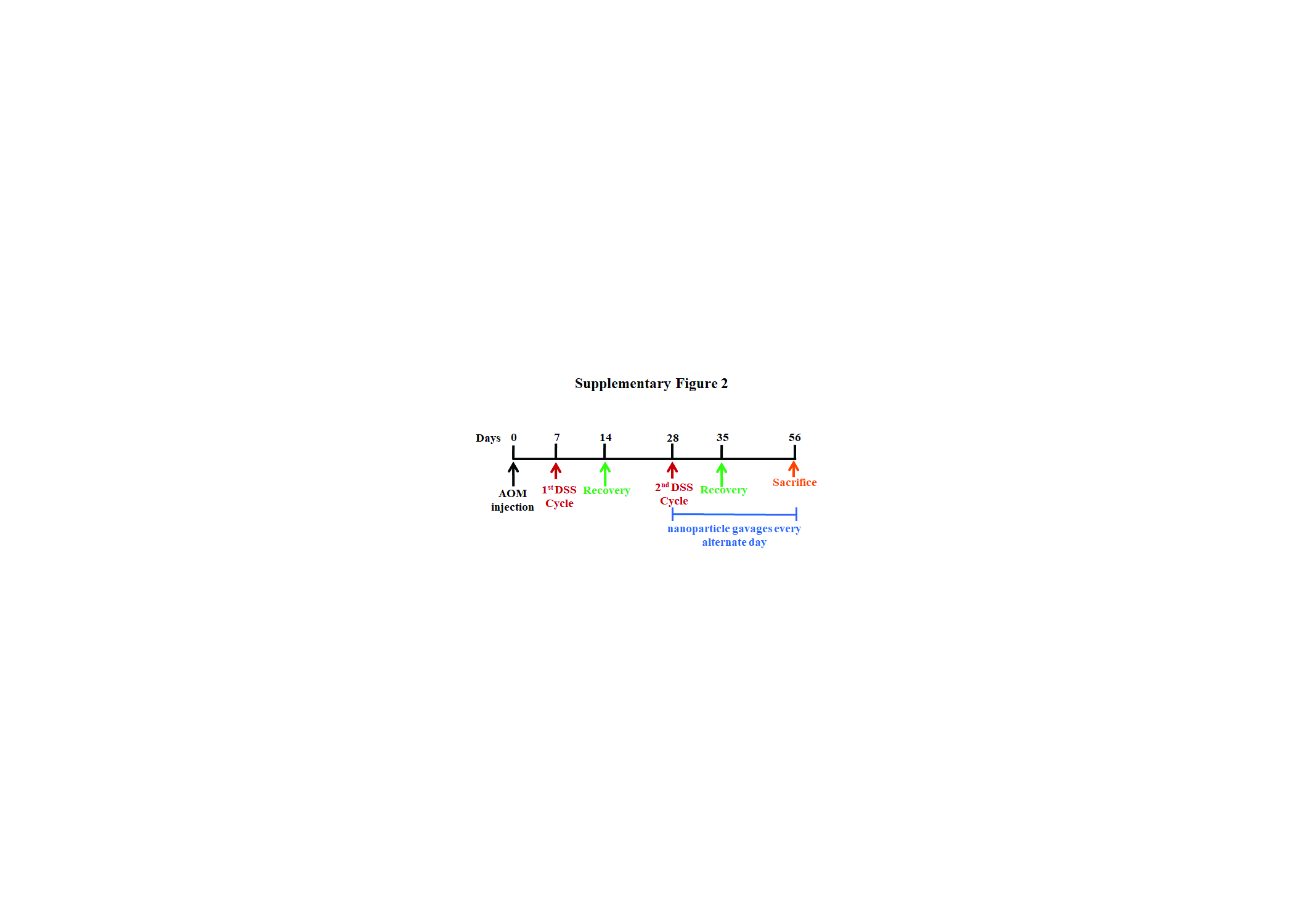

Supplement: Supplementary file 3 — Supplementary Figure 2 [file 41419_2020_2959_MOESM3_ESM.tif]

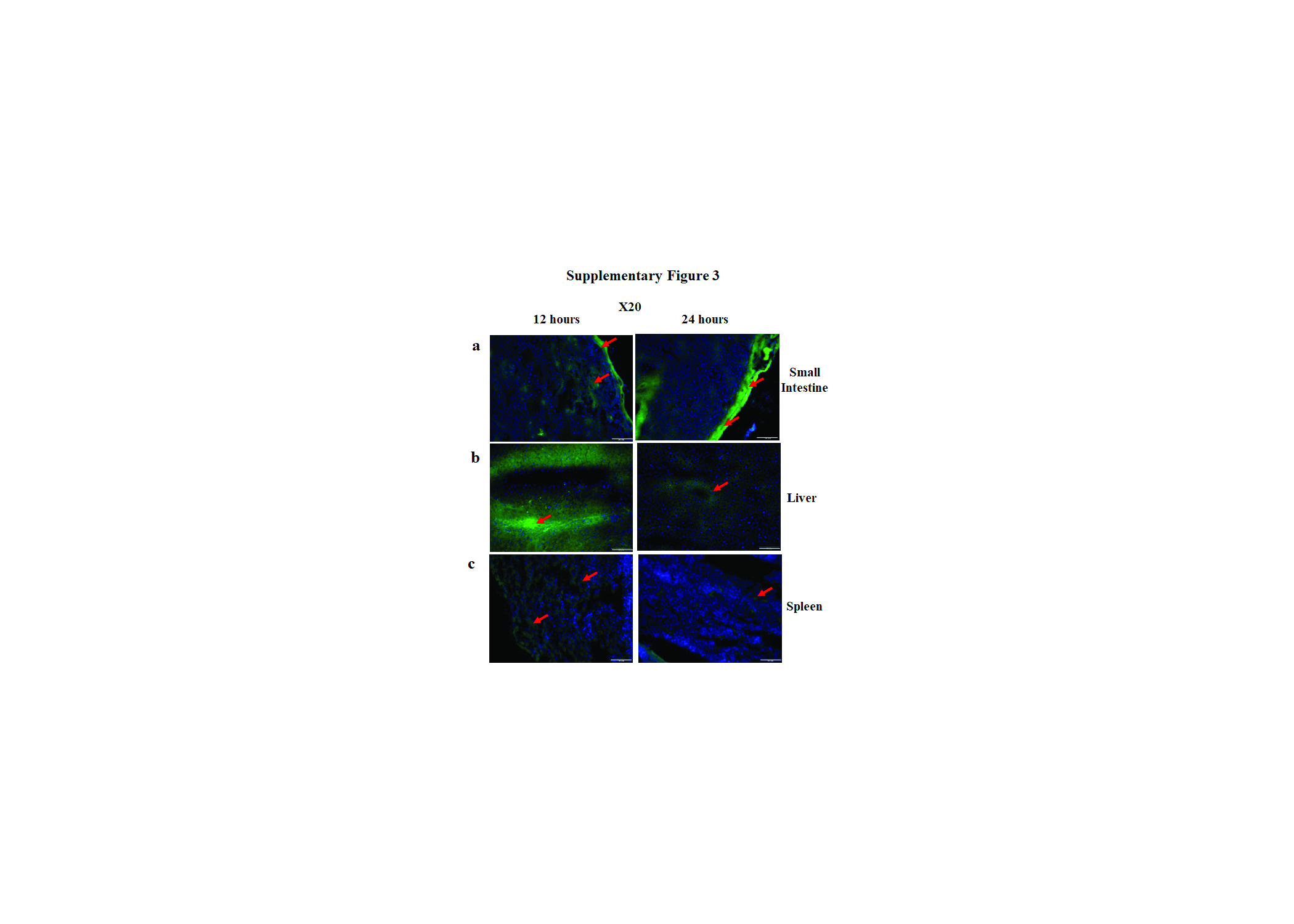

Supplement: Supplementary file 4 — Supplementary Figure 3 [file 41419_2020_2959_MOESM4_ESM.tif]

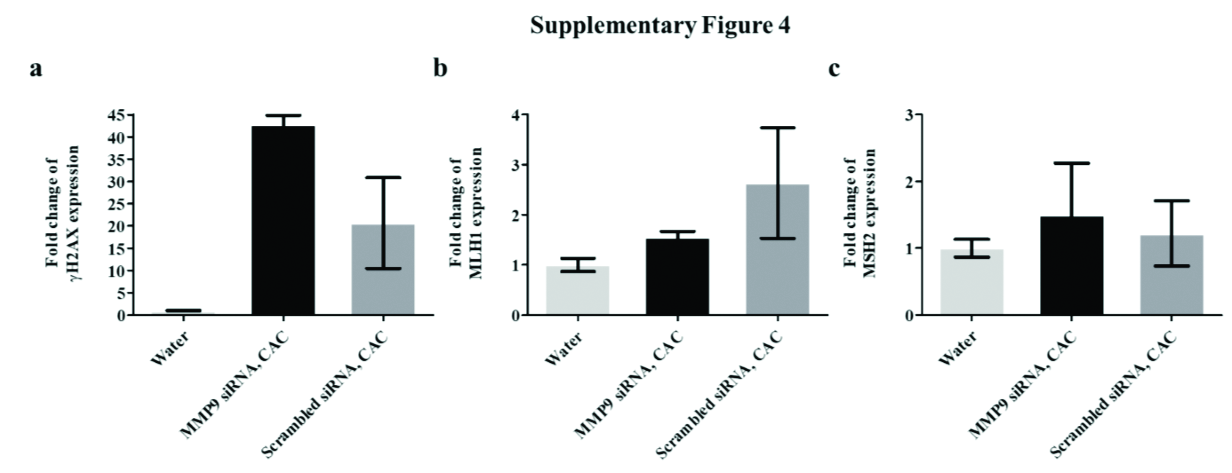

Supplement: Supplementary file 5 — Supplementary Figure 4 [file 41419_2020_2959_MOESM5_ESM.tif]

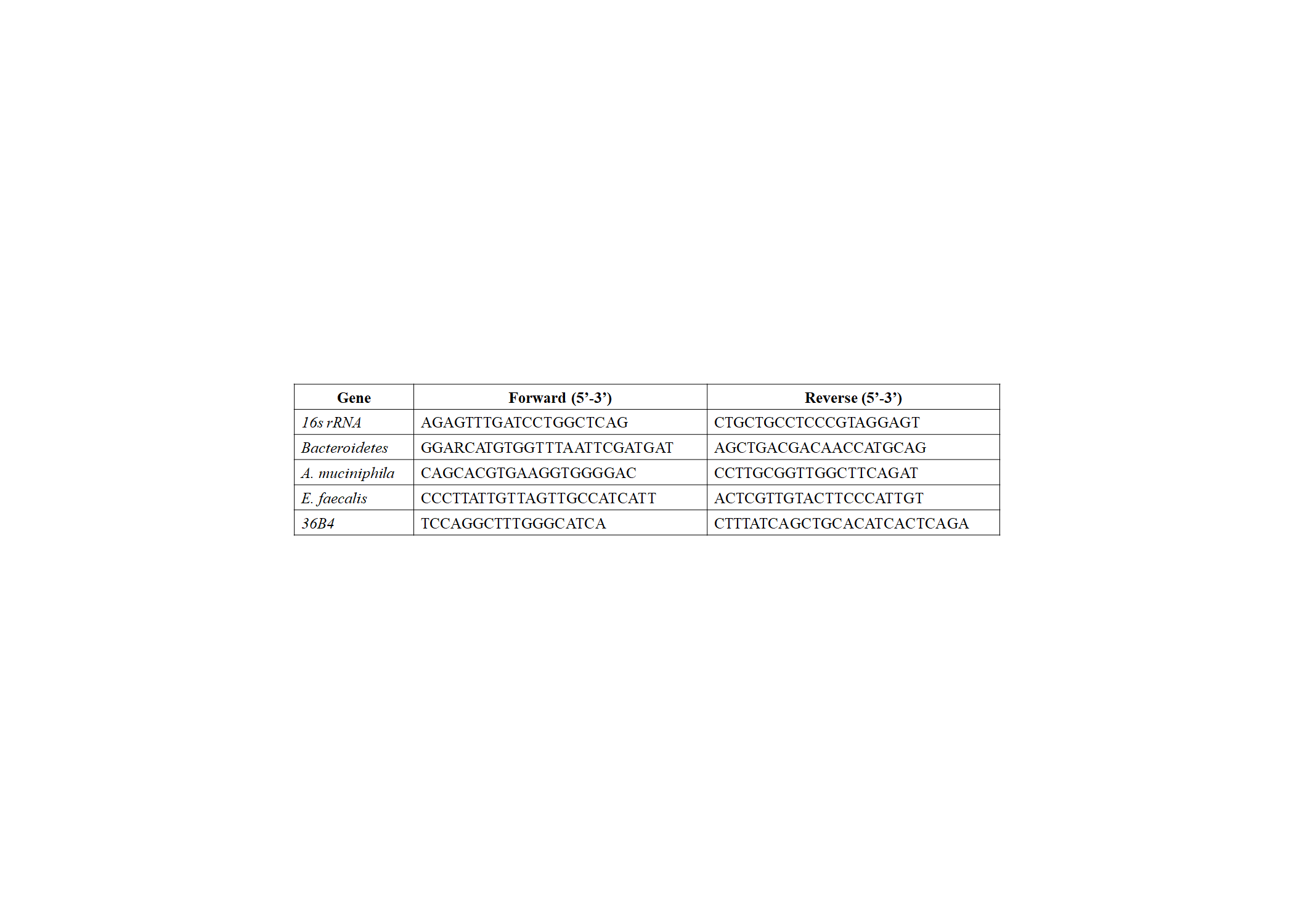

Supplement: Supplementary file 7 — Supplementary Table 1 [file 41419_2020_2959_MOESM7_ESM.tif]
